# Prognostic significance of ZEB1 and ZEB2 in digestive cancers: a cohort-based analysis and secondary analysis

**DOI:** 10.18632/oncotarget.15634

**Published:** 2017-02-23

**Authors:** Huihui Chen, Wei Lu, Chongjie Huang, Kefeng Ding, Dajing Xia, Yihua Wu, Mao Cai

**Affiliations:** ^1^ Department of Oncology, Second Affiliated Hospital, Zhejiang University College of Medicine, Hangzhou, China; ^2^ Department of Anorectal Surgery, The Second Affiliated Hospital and Yuying Children's Hospital of Wenzhou Medical University, Wenzhou, China; ^3^ Department of Toxicology, Zhejiang University School of Public Health, Hangzhou, China; ^4^ Department of Epidemiology and Health Statistics, Zhejiang University School of Public Health, Hangzhou, China

**Keywords:** ZEB family, digestive cancer, prognostic value, cohort-based analysis, secondary analysis

## Abstract

**Background:**

Digestive cancers are common malignancies worldwide, however there are few effective prognostic markers available. In this study we comprehensively investigated the prognostic significance of ZEB1 and ZEB2 in digestive cancers.

**Methods:**

Electronic databases were searched and studies met the selection criteria were included. Study information was recorded and quality assessment was performed according to the REMARK guideline. Hazard ratios and its corresponding 95% confidence intervals were extracted and pooled. Sensitivity analyses, subgroup analyses, cumulative meta-analyses and secondary analyses were also performed to increase the stability and reliability of our results.

**Results:**

24 cohort studies were included in the study. High ZEB1 and ZEB2 levels predicted poor overall survival, meanwhile high ZEB2 levels predicted poor disease free survival for digestive cancer patients. From subgroup analyses we observed ZEB1 was found to be significantly associated with poor overall survival for patients with pancreatic cancer, gastric cancer and colorectal cancer, while ZEB2 was found to be significantly associated with poor overall survival for patients with hepatocellular carcinoma and gastric cancer. Furthermore, by conducting secondary analyses we confirmed both ZEB1 and ZEB2 played important roles in gastric cancer prediction. In addition, we found high ZEB1 and ZEB2 expression were significantly associated with depth of invasion, lymph node metastasis and TNM stage in digestive cancer patients.

**Conclusions:**

The present study validated the prognostic value and clinicopathological association of ZEB1 and ZEB2 in digestive cancers, especially in gastric cancer.

## INTRODUCTION

Digestive cancers are common malignancies featured by their invasiveness and chemo-resistance [1–3], which cause millions of cancer associated deaths worldwide every year [4, 5]. Despite that treatments for digestive cancers have been improved recently, patients’ clinical outcomes still remain unfavorable [4, 6]. Although researchers have paid much effort to identify potential prognostic markers for digestive cancers patients, few tumor markers are put into clinical use routinely [7, 8]. Therefore, it is essential to identify effective prognostic markers in digestive cancers.

Metastatic property, the leading cause of about 90% of cancer patients’ deaths, is the primary characteristic of cancer. Cancer cells could escape from chemotherapy via metastasizing to distant organs, which will lead to poor clinical outcomes. Epithelial mesenchymal transition (EMT) is a process during which cancer cells lose epithelial markers and then increase motility and aggressiveness [9, 10]. Numerous cell signaling pathways are implicated with the induction and maintenance of EMT, such as TGF-beta, Wnt/beta-catenin, Notch and oncogenic Src or Ras signaling [11–14]. Zinc finger E-box binding homeobox 1 (ZEB1, also referred as TCF8, AREB6 and Zfhx1a) and zinc finger E-box binding homeobox 2 (ZEB2, also referred as SIP1, HSPC082 and Zfhx1b) are two ZEB family transcriptional factors involved in the EMT process, which function as either transcriptional activator or repressor depending on their interplay with other transcriptional factors [15, 16]. It is verified that ZEB family could bind to the promoter of CDH1 gene thus repressing the expression of epithelial marker E-cadherin [17–19]. In addition, 3′ untranslated regions of ZEB family are direct target of miR-200 family, whereas promoters of miR-200 family contain highly conserved E-boxes which could be occupied by ZEB1, thus forming a negative self-enforcing feedback loop with miR-200 family [16, 20, 21]. Although accumulating evidences have suggested the oncogenic role of ZEB family, some researchers put forward that ZEB2 can suppress tumor by interacting with retinoblastoma pathway as well [22]. Therefore, further research should be carried out to comprehensively investigate the mechanisms of ZEB family in regulating tumor metastasis.

Various studies have reported aberrant expression of ZEB family members in a multitude of cancers [23–25]. However their clinical relevance in digestive cancers was inconsistent and it remained to be further explored. For example, Zhang et al. found that ZEB1 was a prognostic marker in colorectal cancer and higher expression of ZEB1 weas correlated with liver metastasis [26]. However, Otsuki et al. argued that other EMT markers such as Vimentin rather than ZEB2 predicted decreased overall survival in gastric cancer [27]. Besides, sample sizes of previous studies were relatively small, which may yield unstable results. Hence we performed this cohort-based analysis and secondary analysis to comprehensively investigate the prognostic value of ZEB1 and ZEB2 in digestive cancers.

## RESULTS

### Search results and characteristics of the included studies

**Figure 1:**
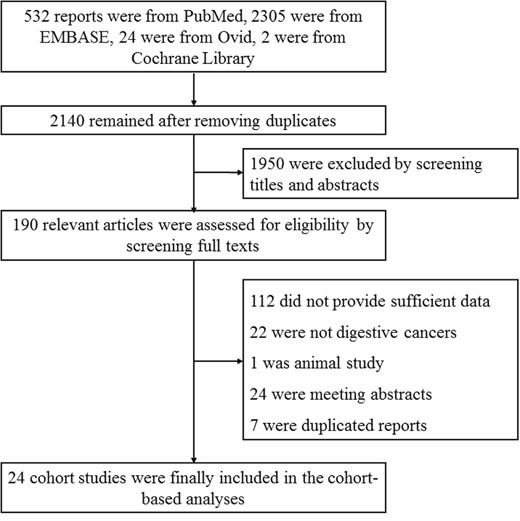
Flow diagram of the study selection process

The initial search in PubMed, EMBASE, Ovid and Cochrane Library electronic databases yielded a total of 2863 articles. After removing 723 duplicated articles, the remaining 2140 articles were carefully screened by scanning titles and abstracts, which resulted in the exclusion of 1950 irrelevant studies. Afterwards 190 relevant studies were assessed for eligibility by scrutinizing full texts including figures and tables. 166 studies were excluded for the following reasons: 112 did not provide sufficient data, 22 were not digestive cancers, 1 was animal study, 24 were meeting abstracts and 7 were duplicated reports (Figure 1).

Finally, 24 cohort studies with 4141 patients were included in the cohort-based analysis, with a mean sample size of 172.5 (ranged from 76 to 690) [23, 24, 26–47]. Features of the 24 studies were listed in Table 1. The period of 24 studies ranged from 2011 to 2016. Among them, Kurahara et al. [34] reported prognostic value of ZEB1 and ZEB2 in pancreatic cancer within 1 article; Okugawa et al. provided prognostic value of ZEB1 [24] and ZEB2 [36] in gastric cancer in 2 articles, which originated from the same cohort; Xia et al. [42] used 2 independent cohort (cohort I and cohort II) to investigate whether ZEB2 expression could predict survival of patients with hepatocellular carcinoma. 11 studies were from China, 9 from Japan, 2 from German, 1 from America and 1 from Thailand. All studies used tissue specimens of patients, while the detection methods mainly focused on immunohistochemistry (IHC) and quantitative real time polymerase chain reaction (qRT-PCR). Types of cancer varied across studies, with 7 studies reported gastric cancer, 5 reported hepatocellular carcinoma, 4 reported colorectal cancer, 4 reported esophageal squamous cells carcinoma, 2 reported cholangiocarcinoma and 2 reported pancreatic cancer. The quality assessment was performed for each individual study according to the REMARK guideline and the results were shown in Table 2.

**Table 1A:** Characteristics of the included studies (ZEB1)

Study (year)	Country	Participants	Follow-up(month)	Age	Specimens	Method	Protein/mRNA	Analysis	Endpoints	Cancer Type	Quality Assessment
Bronsert (2014)	German	59/58 (M/F)	NA	67 (median)	tissue	IHC	protein	multivariable	OS	pancreatic cancer	14
Goscinski (2015)	China	92/59 (M/F)	NA	33-73	tissue	IHC	protein	univariable	OS	esophageal squamous cell carcinoma	12
Hara (2014)	Japan	79/14 (M/F)	46 (median)	64 (mean)	tissue	IHC	protein	univariable	OS	esophageal squamous cell carcinoma	14
Hashiguchi (2013)	Japan	85/23 (M/F)	48.4 (median)	65.3 (mean)	tissue	IHC	protein	multivariable	OS	hepatocellular carcinoma	16
Kurahara (2012)	Japan	52/24 (M/F)	NA	67 (median)	tissue	IHC	protein	univariable	OS	pancreatic cancer	13
Murai (2014)	Japan	83/33 (M/F)	37 (median)	64 (mean)	tissue	qRT-PCR	mRNA	univariable	OS	gastric cancer	16
Okugawa (2012)	Japan	106/28 (M/F)	23 (median)	67 (mean)	tissue	qRT-PCR	mRNA	multivariable	OS	gastric cancer	14
Singh (2011)	America	136/114 (M/F)	0.4-142.6	64.6 (mean)	tissue	gene chip	mRNA	univariable	OS	colonrectal cancer	9
Terashita (2016)	Japan	63/39 (M/F)	35 (median)	NA	tissue	IHC	protein	multivariable	OS	cholangiocarcinoma	16
Wu (2016)	China	145 (total)	47.7 (median)	NA	tissue	IHC	protein	multivariable	OS, RFS	colonrectal cancer	17
Yang X. (2014)	China	68/32 (M/F)	32 (median)	50 (median)	tissue	IHC	protein	univariable	OS	esophageal squamous cell carcinoma	16
Zhang (2013)	China	50/42 (M/F)	NA	62 (mean)	tissue	qRT-PCR	mRNA	multivariable	OS	colonrectal cancer	16
Zhou L. (2016)	China	172/89 (M/F)	8-110	NA	tissue	IHC	protein	multivariable	OS	gastric cancer	16
Zhou Y. (2012)	China	98/12 (M/F)	NA	54 (median)	tissue	western blot	protein	multivariable	OS, DFS	hepatocellular carcinoma	13
Zhou Y. (2012)	China	98/12 (M/F)	NA	54 (median)	tissue	western blot	protein	multivariable	OS, DFS	hepatocellular carcinoma	13
Zhou Y. (2012)	China	98/12 (M/F)	NA	54 (median)	tissue	western blot	protein	multivariable	OS, DFS	hepatocellular carcinoma	13

M: male; F: female; NA: not available; IHC: immunohistochemistry; qRT-PCR: quantitative real time polymerase chain reaction; OS: overall survival; RFS: recurrence free survival; DFS: disease free survival.

**Table 1B:** Characteristics of the included studies (ZEB2)

Study (year)	Country	Participants	Follow-up(month)	Age	Specimens	Method	Protein/mRNA	Analysis	Endpoints	Cancer Type	Quality Assessment
Cai (2012)	China	220/28 (M/F)	26.0 (median)	47.8 (mean)	tissue	IHC	protein	multivariabe	OS	hepatocellular carcinoma	17
Dai (2012)	China	50/26 (M/F)	40 (median)	53.8 (mean)	tissue	IHC	protein	Univariable	OS	gastric cancer	14
Kahlert (2011)	German	121/54 (M/F)	124 (median)	NA	tissue	IHC	protein	multivariabe	DFS	colorectal cancer	14
Kurahara (2012)	Japan	52/24 (M/F)	NA	67 (median)	tissue	IHC	protein	univariable	OS	pancreatic cancer	13
Okugawa (2013)	Japan	106/28 (M/F)	23 (median)	67 (mean)	tissue	qRT-PCR	mRNA	multivariabe	OS	gastric cancer	15
Otsuki (2011)	Japan	84/22 (M/F)	48 (median)	NA	tissue	qRT-PCR	mRNA	univariable	DFS, RFS	gastric cancer	15
Sun (2015)	Chian	192/69 (M/F)	50 (median)	59 (mean)	tissue	IHC	protein	univariable	OS, DFS	gastric cancer	17
Techasen (2014)	Thailand	149/66 (M/F)	NA	21-82	tissue	IHC, qRT-PCR	protein, mRNA	univariable	OS	cholangiocarcinoma	13
Xia-cohort I (2014)	China	581/109 (M/F)	4-96	51.8 (mean)	tissue	IHC	protein	univariable	OS	hepatocellular carcinoma	15
Xia-cohort II (2014)	China	256/56 (M/F)	4-96	51.9 (mean)	tissue	IHC	protein	univariable	OS	hepatocellular carcinoma	15
Yang Z. (2015)	China	79/13 (M/F)	NA	NA	tissue	IHC	protein	univariable	OS	hepatocellular carcinoma	11
Yoshida (2015)	Japan	100/11 (M/F)	NA	64.3 (mean)	tissue	IHC	protein	univariable	OS, DFS	esophageal squamous cell carcinoma	12
Yoshida (2015)	Japan	100/11 (M/F)	NA	64.3 (mean)	tissue	IHC	protein	univariable	OS, DFS	esophageal squamous cell carcinoma	12
Yoshida (2015)	Japan	100/11 (M/F)	NA	64.3 (mean)	tissue	IHC	protein	univariable	OS, DFS	esophageal squamous cell carcinoma	12

M: male; F: female; NA: not available; IHC: immunohistochemistry; qRT-PCR: quantitative real time polymerase chain reaction; OS: overall survival; RFS: recurrence free survival; DFS: disease free survival.

**Table 2A:** Quality assessment according to the REMARK guideline (ZEB1)

Study	Q1	Q2	Q3	Q4	Q5	Q6	Q7	Q8	Q9	Q10	Q11	Q12	Q13	Q14	Q15	Q16	Q17	Q18	Q19	Q20	Total
Bronsert (2014)	1	1	1	1	1	1	0	0	0	1	1	1	1	0	1	0	0	1	1	1	14
Goscinski (2015)	1	1	1	1	1	0	0	0	0	1	1	0	0	1	1	0	0	1	1	1	12
Hara (2014)	1	1	1	1	1	1	0	1	0	1	1	0	0	0	1	1	0	1	1	1	14
Hashiguchi (2013)	1	1	1	1	1	1	0	0	0	1	1	0	1	1	1	1	1	1	1	1	16
Kurahara (2012)	1	1	1	1	1	0	0	0	0	1	1	0	1	1	1	0	0	1	1	1	13
Murai (2014)	1	1	1	1	1	1	0	0	0	1	1	0	1	1	1	1	1	1	1	1	16
Okugawa (2012)	1	1	1	1	1	1	0	1	0	1	0	0	1	0	1	1	0	1	1	1	14
Singh (2011)	1	1	1	1	1	0	0	0	0	1	0	0	0	0	0	0	0	1	1	1	9
Terashita (2016)	1	1	1	1	1	1	0	1	0	1	1	0	1	1	0	1	1	1	1	1	16
Wu (2016)	1	1	1	1	1	1	1	1	0	1	1	0	0	1	1	1	1	1	1	1	17
Yang X. (2014)	1	1	1	1	1	1	1	1	0	1	1	0	1	1	1	0	0	1	1	1	16
Zhang (2013)	1	1	1	1	1	0	0	1	0	1	1	0	1	1	1	1	1	1	1	1	16
Zhou L. (2016)	1	1	1	1	1	1	1	1	0	1	1	0	1	1	0	1	0	1	1	1	16
Zhou Y. (2012)	1	1	1	1	1	0	0	0	0	1	1	0	1	1	0	1	0	1	1	1	13

**Table 2B:** Quality assessment according to the REMARK guideline (ZEB2)

Study	Q1	Q2	Q3	Q4	Q5	Q6	Q7	Q8	Q9	Q10	Q11	Q12	Q13	Q14	Q15	Q16	Q17	Q18	Q19	Q20	Total
Cai (2012)	1	1	1	1	1	1	0	1	0	1	1	0	1	1	1	1	1	1	1	1	17
Dai (2012)	1	1	1	1	1	1	0	0	0	1	1	0	1	1	1	0	0	1	1	1	14
Kahlert (2011)	1	1	1	1	1	1	0	1	0	1	1	0	0	0	1	1	0	1	1	1	14
Kurahara (2012)	1	1	1	1	1	0	0	0	0	1	1	0	1	1	1	0	0	1	1	1	13
Okugawa (2013)	1	1	1	1	1	1	0	1	0	1	0	0	1	1	1	1	0	1	1	1	15
Otsuki (2011)	1	1	1	1	1	1	0	0	0	1	1	0	1	1	1	1	0	1	1	1	15
Sun (2015)	1	1	1	1	1	1	1	0	0	1	1	0	1	1	1	1	1	1	1	1	17
Techasen (2014)	1	1	1	1	1	0	0	0	0	1	1	0	1	1	1	0	0	1	1	1	13
Xia-cohort I (2014)	1	1	1	1	1	1	1	1	0	1	1	0	1	1	0	0	0	1	1	1	15
Xia-cohort II (2014)	1	1	1	1	1	1	1	1	0	1	1	0	1	1	0	0	0	1	1	1	15
Yang Z. (2015)	1	1	0	1	1	0	0	0	0	1	1	0	1	1	0	0	0	1	1	1	11
Yoshida (2015)	1	1	1	1	1	0	0	0	0	1	1	0	1	1	0	0	0	1	1	1	12

### High ZEB1 and ZEB2 levels predicted poor overall survival in digestive cancers

**Figure 2:**
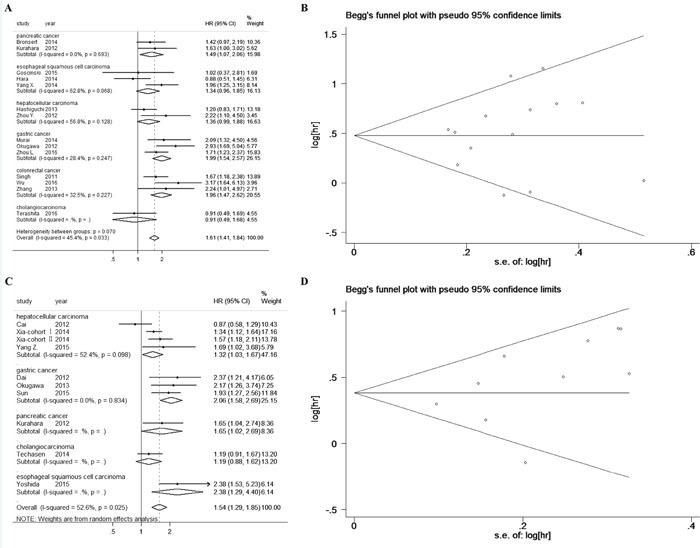
High ZEB1 and ZEB2 levels predicted poor overall survival in digestive cancers **A**. Forest plot of HR for the association between ZEB1 expression and overall survival in patients with digestive cancers. **B**. Funnel plot for the association between ZEB1 expression and overall survival in patients with digestive cancers. **C**. Forest plot of HR for the association between ZEB2 expression and overall survival in patients with digestive cancers. **D**. Funnel plot for the association between ZEB2 expression and overall survival in patients with digestive cancers.

The impact of tissue ZEB1 and ZEB2 expression on overall survival (OS) was investigated respectively. 14 studies reported the OS of 1855 patients according to ZEB1 expression and 10 studies reported the OS of 2215 patients according to ZEB2 expression. The heterogeneity test revealed that there was no significant heterogeneity in the 14 studies for ZEB1 (I^2^=45.4%, *p*=0.033), while significant heterogeneity existed in the 10 studies for ZEB2 (I^2^=52.6%, *p*=0.025). Therefore we adopted the fixed-effect model and the random-effect model for ZEB1 and ZEB2 respectively. As shown in Figure 2A and Figure 2C, pooled analyses showed that elevated ZEB1 expression predicted unfavorable OS in digestive cancer patients (pooled HR: 1.610, 95% CI: 1.412-1.835, *p*<0.001), so did ZEB2 (pooled HR: 1.543, 95% CI: 1.288-1.848, *p*<0.001). Begg's funnel plot and Egger's test were carried to assess potential publication bias. The Begg's funnel plots were symmetrical, indicating that there was no significant publication bias, with *p*_Begg_=0.743 and *p*_Egger_=0.556 for ZEB1 (Figure 2B) and *p*_Begg_=0.474 and *p*_Egger_=0.142 for ZEB2 (Figure 2D).

### Sensitivity analyses, subgroup analyses and cumulative meta-analysis

**Figure 3:**
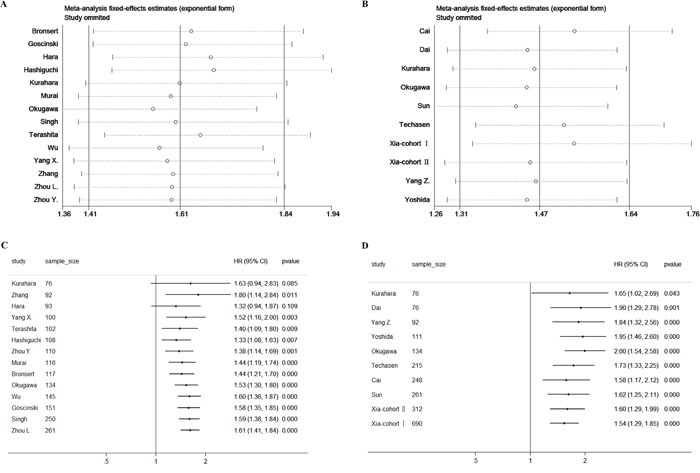
Sensitivity analyses by sequentially omitting single study for A. ZEB1 and B. ZEB2 Cumulative meta-analysis was performed according to sample size for **C**. ZEB1 and **D**. ZEB2, and the studies were added one at a time to pool the results sequentially.

Sensitivity analyses were performed by sequentially omitting single study to assess the stability of the pooled results. As shown in Figure 3A and Figure 3B, no individual study changed pooled HR significantly.

We further performed subgroup analyses according to cancer type, country of origin, protein/mRNA, quality assessment score and sample size (Table 3). ZEB1 was found to be significantly associated with poor OS for patients with pancreatic cancer (pooled HR: 1.487, 95% CI: 1.071-2.064, *p*=0.018), gastric cancer (pooled HR: 1.990, 95% CI: 1.540-2.573, *p*<0.001) and colorectal cancer (pooled HR: 1.961, 95% CI: 1.468-2.619, *p*<0.001), while ZEB2 was found to be significantly associated with poor OS for patients with hepatocellular carcinoma (pooled HR: 1.315, 95% CI: 1.033-1.674, *p*=0.026) and gastric cancer (pooled HR: 2.063, 95% CI: 1.582-2.691, *p*<0.001). However, ZEB1 did not predict poor OS for patients with esophageal squamous cell carcinoma (pooled HR: 1.338, 95% CI: 0.965-1.854, *p*=0.081) and hepatocellular carcinoma (pooled HR: 1.364, 95% CI: 0.989-1.881, *p*=0.059). Subgroup analyses aiming at other cancer types could not be conducted due to the limited study number.

**Table 3:** Subgroup analyses

	ZEB1							ZEB2						
	pooled HR	95% CI	p	heterogeneity		*p*_Begg_	*p*_Egger_	pooled HR	95% CI	p	heterogeneity		*p*_Begg_	*p*_Egger_
				I^2^ (%)	p						I^2^ (%)	p		
cancer type														
pancreatic cancer	1.487	(1.071, 2.064)	0.018	0.0	0.693	1.000	-	-	-	-	-	-	-	-
esophageal squamous cell carcinoma	1.338	(0.965, 1.854)	0.081	62.8	0.068	1.000	0.731	-	-	-	-	-	-	-
hepatocellular carcinoma	1.364	(0.989, 1.881)	0.059	56.8	0.128	1.000	-	1.315	(1.033, 1.674)	0.026	52.4	0.098	1.000	0.886
gastric cancer	1.990	(1.540, 2.573)	<0.001	28.4	0.247	1.000	0.432	2.063	(1.582, 2.691)	<0.001	0.0	0.834	0.296	0.093
colonrectal cancer	1.961	(1.468, 2.619)	<0.001	32.5	0.227	1.000	0.393	-	-	-	-	-	-	-
country														
China	1.926	(1.547, 2.399)	<0.001	0.0	0.476	1.000	0.724	1.493	(1.180, 1.889)	0.001	60.2	0.028	1.000	0.551
Japan	1.443	(1.002, 2.078)	0.049	65.6	0.013	1.000	0.601	1.986	(1.453, 2.714)	<0.001	0.0	0.609	0.296	0.209
protein/mRNA														
protein	1.488	(1.194, 1.854)	<0.001	46.1	0.054	0.858	0.929	1.500	(1.247, 1.805)	<0.001	52.7	0.031	0.754	0.240
mRNA	2.013	(1.563, 2.592)	<0.001	0.0	0.392	0.734	0.345	1.630	(0.924, 2.875)	0.092	52.9	0.145	1.000	-
quality assessment														
score >=15	1.693	(1.290, 2.222)	<0.001	50.1	0.062	0.764	0.418	1.472	(1.142, 1.898)	0.003	66.7	0.017	0.806	0.693
score <15	1.586	(1.196, 2.104)	0.001	48.8	0.069	1.000	0.966	1.668	(1.246, 2.234)	0.001	39.7	0.156	0.806	0.023
sample size														
large (>=100)	1.702	(1.394, 2.078)	<0.001	43.8	0.059	0.640	0.535	1.478	(1.194, 1.829)	<0.001	62.5	0.014	0.548	0.391
small (<100)	1.397	(0.820, 2.379)	0.218	55.9	0.103	0.296	0.446	1.840	(1.324, 2.555)	<0.001	0.0	0.636	1.000	0.635

Since most of the studies were conducted in China or Japan, we also stratified studies depending on the country of origin. We detected a significant association between ZEB family member expression and poor OS for patients with digestive cancers in China (pooled HR for ZEB1: 1.926, 95% CI: 1.547-2.399, *p*<0.001; pooled HR for ZEB2: 1.493, 95% CI: 1.180-1.889, *p*=0.001) or Japan (pooled HR for ZEB1: 1.443, 95% CI: 1.002-2.078, *p*=0.049; pooled HR for ZEB2: 1.986, 95% CI: 1.453-2.714, *p*<0.001). In addition, we further investigated whether protein and mRNA of ZEB family had the same prognostic value in digestive cancers. We found high ZEB1 and ZEB2 protein was associated with poor OS consistently (pooled HR for ZEB1: 1.488, 95% CI: 1.194-1.854, *p*<0.001; pooled HR for ZEB2: 1.500, 95% CI: 1.247-1.805, *p*<0.001), however high ZEB1 mRNA predicted poor OS while high ZEB2 did not show the same effect, even though it had the tendency (pooled HR for ZEB1: 2.013, 95% CI: 1.563-2.592, *p*<0.001; pooled HR for ZEB2: 1.630, 95% CI: 0.924-2.875, *p*=0.092). We divided studies into high quality group and low quality group according to quality assessment score (high quality group: score>=15; low quality group: score<15). Both ZEB1 and ZEB2 predicted unfavorable OS in high quality group (pooled HR for ZEB1: 1.693, 95% CI: 1.290-2.222, *p*<0.001; pooled HR for ZEB2: 1.472, 95% CI: 1.142-1.898, *p*=0.003) and low quality group (pooled HR for ZEB1: 1.586, 95% CI: 1.196-2.104, *p*=0.001; pooled HR for ZEB2: 1.668, 95% CI: 1.246-2.234, *p*=0.001).

To be noticed, when performing subgroup analyses stratified by sample size, we detected that ZEB1 and ZEB2 was significantly associated with poor OS in large sample size group (pooled HR for ZEB1: 1.702, 95% CI: 1.394-2.078, *p*<0.001; pooled HR for ZEB2: 1.478, 95% CI: 1.194-1.829, *p*<0.001), while ZEB1 failed to achieve statistical significance in small sample size group (pooled HR for ZEB1: 1.397, 95% CI: 0.820-2.379, *p*=0.218; pooled HR for ZEB2: 1.840, 95% CI: 1.324-2.555, *p*<0.001). Therefore we further performed cumulative meta-analysis by sorting of the included studies according to the sample size. In accordance with subgroup analyses, we observed that statistical significance was reached after including large sample size studies (sample size >= 100) for ZEB1, while the pooled HR has already met statistical significance since including small sample size studies (sample size < 100) for ZEB2, as shown in Figure 3C and Figure 3D. This finding implied that including studies with large sample size in the meta-analysis contributed to more stable results.

### Secondary analyses confirmed prognostic significance of ZEB1 and ZEB2 for patients with gastric cancer

**Figure 4:**
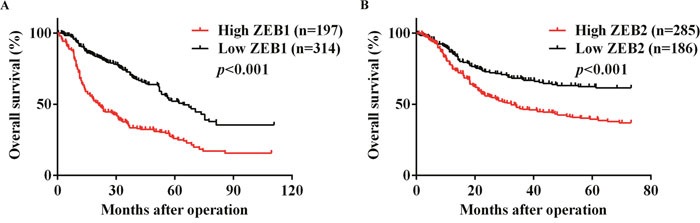
Reconstructed Kaplan Meier survival curves for overall survival of gastric cancer patients according to tissue A. ZEB1 and B. ZEB2 level

Since pooling studies with small sample size might bring about unstable results, secondary analyses utilizing time-to-event patient data were carried out to enlarge sample size. We only performed secondary analyses in gastric cancer, because only in gastric cancer did both ZEB1 and ZEB2 have significant prognostic value, and studies concerning gastric cancer provided adequate data for secondary analyses. Guyot's method was used to acquire time-to-event patient data from Kaplan-Meier survival curves. Survival curves of 3 studies from Murai [35], Okugawa [24] and Zhou L. [46] were extracted for ZEB1, and survival curves of 3 studies from Dai [29], Okugawa [36] and Sun [38] were extracted for ZEB2, respectively. The reconstructed survival curves were displayed in Figure 4A and Figure 4B, which confirmed prognostic significance of ZEB1 and ZEB2 for patients with gastric cancer (HR for ZEB1: 2.305, 95% CI: 2.113-3.465, log-rank *p*<0.001; HR for ZEB2: 1.927, 95% CI: 1.416-2.382, log-rank *p*<0.001).

### High ZEB2 level predicted disease free survival in digestive cancers

**Figure 5:**
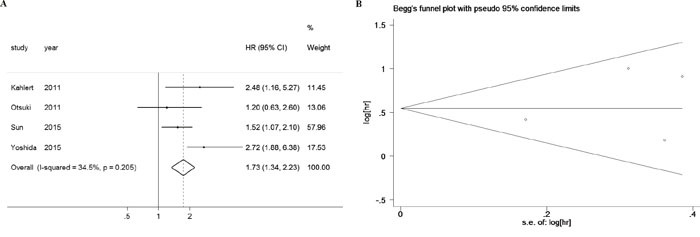
High ZEB2 levels predicted poor disease free survival in digestive cancers **A**. Forest plot of HR for the association between ZEB2 expression and disease free survival in patients with digestive cancers. **B**. Funnel plot for the association between ZEB2 expression and disease free survival in patients with digestive cancers.

The impact of tissue ZEB2 expression on disease free survival (DFS) in digestive cancers was also investigated, whereas only Zhou Y. et al. [47] reported the association between tissue ZEB1 expression and DFS so it could not be performed. The heterogeneity test revealed that there was no significant heterogeneity between studies (I^2^=34.5%, *p*=0.205). The fixed effect model was adopted and the pooled analyses showed that elevated ZEB2 expression predicted poor DFS in digestive cancer patients (pooled HR: 1.726, 95% CI: 1.336-2.230, *p*<0.001), as shown in Figure 5A. The Begg's funnel plots were symmetrical (Figure 5B), indicating that there was no significant publication bias, with *p*_Begg_=0.734 and *p*_Egger_=0.554. We did not perform further subgroup analyses due to the limited study number.

### The association between increased ZEB family expression and clinicopathological features in digestive cancer patients

The above results have demonstrated the prognostic significance of ZEB family, and we further investigated the association between increased ZEB family expression and clinicopathological features in digestive cancer patients. From the 24 included cohort studies, there were 16 studies providing sufficient clinicopathological data of digestive cancer patients for analyses [26, 29, 32–35, 38–40, 43–47]. In addition to that, another 1 previous excluded non-cohort study was retrieved because it reported the relationship between ZEB1 expression in gastric cancer tissues and clinicopathological characteristics [48]. As shown in Table 4, we observed that high ZEB family expression were not associated with age (pooled OR for ZEB1: 0.741, 95% CI: 0.442-1.243, *p*=0.256; pooled OR for ZEB2: 1.155, 95% CI: 0.854-1.561, *p*=0.349) or gender (pooled OR for ZEB1: 0.902, 95% CI: 0.678-1.200, *p*=0.479; pooled OR for ZEB2: 1.010, 95% CI: 0.746-1.369, *p*=0.948). Interestingly, high ZEB1 expression was significantly associated with large tumor size (pooled OR for ZEB1: 1.571, 95% CI: 1.162-2.124, *p*=0.003; pooled OR for ZEB2: 1.318, 95% CI: 0.888-1.956, *p*=0.171) and poor differentiation (pooled OR for ZEB1: 2.428, 95% CI: 1.644-3.578, *p*<0.001; pooled OR for ZEB2: 1.068, 95% CI: 0.159-7.146, *p*=0.946), while ZEB2 did not show the same effect. What's more, both ZEB1 and ZEB2 were found to be significantly associated with depth of invasion (pooled OR for ZEB1: 2.423, 95% CI: 1.311-4.478, *p*=0.005; pooled OR for ZEB2: 2.187, 95% CI: 1.009-4.743, *p*=0.047), lymph node metastasis (pooled OR for ZEB1: 3.136, 95% CI: 2.278-4.317, *p*<0.001; pooled OR for ZEB2: 2.360, 95% CI: 1.701-3.276, *p*<0.001) and TNM stage (pooled OR for ZEB1: 4.194, 95% CI: 2.449-7.183, *p*<0.001; pooled OR for ZEB2: 3.169, 95% CI: 2.079-4.830, *p*<0.001). There were significant heterogeneity between studies regarding age, depth of invasion and TNM stage for ZEB1, while differentiation and depth of invasion for ZEB2. However, further subgroup analyses were not applicable for the relationship between ZEB family expression and clinicopathological features of digestive cancer patients because of the limited number of studies. Besides, Begg's test and Egger's test both showed the absence of potential publication bias.

**Table 4:** Association between increased ZEB family expression and clinicopathological features in digestive cancer patients

	ZEB1							ZEB2						
	pooled OR	95% CI	p	heterogeneity		*p*_Begg_	*p*_Egger_	pooled OR	95% CI	p	heterogeneity		*p*_Begg_	*p*_Egger_
				I^2^ (%)	p						I^2^ (%)	p		
age (old vs young)^1^	0.741	(0.442, 1.243)	0.256	59.1	0.032	1.000	0.735	1.155	(0.854, 1.561)	0.349	44.5	0.125	0.806	0.619
gender (male vs female)	0.902	(0.678, 1.200)	0.479	46.4	0.061	0.466	0.127	1.010	(0.746, 1.369)	0.948	0.0	0.631	1.000	0.715
tumor size (large vs small)^2^	1.571	(1.162, 2.124)	0.003	0.0	0.937	0.902	0.629	1.318	(0.888, 1.956)	0.171	0.0	0.712	1.000	0.616
differentiation (poor vs moderate+well)	2.428	(1.644, 3.578)	<0.001	22.9	0.268	0.806	0.617	1.068	(0.159, 7.146)	0.946	93.7	<0.001	0.296	0.182
depth of invasion (T3+T4 vs T1+T2 or T4 vs T1+T2+T3)	2.423	(1.311, 4.478)	0.005	50.9	0.07	0.260	0.247	2.187	(1.009, 4.743)	0.047	61.0	0.053	1.000	0.646
lymph node metastasis (positive vs negative)	3.136	(2.278, 4.317)	<0.001	6.8	0.376	0.764	0.932	2.360	(1.701, 3.276)	<0.001	28.4	0.232	0.462	0.021
TNM stage (III+IV vs I+II or IV vs I+II+III)	4.194	(2.449, 7.183)	<0.001	57.2	0.029	0.764	0.508	3.169	(2.079, 4.830)	<0.001	0.0	0.610	1.000	0.094

1: The cut-off value of age was various across studies.

2: Tumor size was measured according to diameter or volume across studies.

## DISCUSSION

Identifying potential prognostic markers for digestive cancer patients is necessary as these markers will help provide valuable information for clinical scientists. In recent years, the association between ZEB family and patient clinical outcome has been reported in various cancers, despite that the results were inconsistent across studies [23–47]. We conducted this first cohort-based analysis and secondary analysis focusing on the prognostic value of ZEB family members in digestive cancers. Our results implied that both high ZEB1 and ZEB2 expression predicted poor OS in patients with digestive cancers. Specifically, ZEB1 was found to be significantly associated with poor OS for pancreatic cancer, gastric cancer and colorectal cancer patients, while ZEB2 was found to be significantly associated with poor OS for hepatocellular carcinoma and gastric cancer patients. The reconstructed survival curves utilizing time-to-event data confirmed the prognostic value of ZEB family in gastric cancer as well. In addition, we also observed ZEB2 expression predicted poor DFS in patients with digestive cancers. Finally, the results showed high ZEB1 expression was significantly associated with tumor size, differentiation, depth of invasion, lymph node metastasis and TNM stage, while high ZEB2 expression was significantly associated with depth of invasion, lymph node metastasis and TNM stage in digestive cancer patients.

Explanations for the prognostic value of ZEB family might require multiple mechanisms. First, classical theory that ZEB family members suppress E-cadherin expression and induce EMT is widely accepted [17, 19]; Second, ZEB family could form a complex regulatory network with p53 family members and their downstream targets, thus modulating cell cycle progression and apoptosis [49, 50]. Besides, ZEB1 has been reported to help cancer cells develop resistance to radiation via stabilizing CHK1 [51], while ZEB2 could protect cancer cells from UV or cisplatin induced apoptosis [52]. Overall, digestive cancer patients with high expression of ZEB family tend to have metastatic tumors or acquire resistance to chemotherapy or radiotherapy, thus leading to poor survival outcomes.

We found that ZEB2 did not always have the same prognostic significance and clinicopathological association as ZEB1, especially when performing subgroup analyses according to cancer type. One possible interpretation is that ZEB family may have different expression profiles in various cancer tissues. Besides, different signaling pathways are involved and ZEB2 was reported to act as a tumor suppressor in some cancers via mediating the TGF-beta regulated repression of hTERT [53] and interacting with retinoblastoma pathway [54]. In the present study, we observed the prognostic value of ZEB family was most effective in gastric cancer, as verified by our secondary analyses as well. There are several reasons for that. First, Murai et al. [35] found that compared with epithelial status, mesenchymal status predicted poor OS in gastric cancer, which made it logical and reasonable considering ZEB family's EMT promoting role. Furthermore, high ZEB1 expression was an independent indicator of peritoneal dissemination, which was responsible for the majority of mortality in gastric cancer patients [24]. Apart from ZEB family protein level, Yabusaki et al. [55] found that ZEB1 mRNA in peritoneal washing was associated with poor survival and clinicopathological features, which may account for that ZEB1 mRNA, but not ZEB2 mRNA, predicted poor OS for digestive cancer patients. Besides, high ZEB2 expression was strongly associated with lactate dehydrogenase A (LDHA) expression in gastric cancer [38], and LDHA was a crucial enzyme in the final step of the Warburg effect, through which high rate of glycolysis was executed in cancer cells [56]. Still, further studies will be necessary to explore the molecular mechanisms and clinical significance of ZEB family in digestive cancers, especially in gastric cancer.

It is noteworthy that our study had numerous strengths. To our limited knowledge, we conducted a first systematic literature search and applied a scientific approach to comprehensively investigate the prognostic significance of ZEB family in digestive cancers. The included studies were all cohort studies of high methodological quality. Sensitivity analyses were performed to increase the stability and reliability of the pooled results, and we also further investigated the association between ZEB family and survival outcome in various subgroups. In addition, we performed secondary analyses adopting enlarged sample size to confirm the prognostic significance of ZEB family in gastric cancer. After validating the prognostic value of ZEB family, we further investigated the association between ZEB family expression and clinicopathological features in digestive cancer patients. The methods of this study were rigorous and were based on guidelines for conducting the present study.

Still, this cohort-based analysis was limited in some aspects as well. First, the number of studies was relatively small thus sufficient subgroup analyses according to cancer type could not be performed. Second, we did not perform pooled analyses for the association between tissue ZEB1 expression and DFS because only 1 study reported it. Third, the majority of the studies were conducted in China or Japan, so the conclusions should be taken cautiously when applied for other ethnic populations. We suggested more cohort studies concerning a specific type of digestive cancer were needed to further identify the prognostic value of ZEB family and follow-up endpoints such as DFS or RFS should also be recorded. Although the incidences of some digestive cancers were relatively low in western countries, the ethnic composition of patients should be diverse. Finally, future cohort studies should recruit more patients to enlarge sample size, which will yield more stable and reliable results.

In conclusion, the present cohort-based analysis validated the prognostic value and clinicopathological association of ZEB family in digestive cancers, especially in gastric cancer.

## MATERIALS AND METHODS

### Search strategy

We performed a systematic literature search in four electronic databases: PubMed, EMBASE, Ovid and Cochrane Library. The search strategy was as follows: ((“ZEB1” OR “AREB6” OR “BZP” OR “DELTAEF1” OR “FECD6” OR “NIL2A” OR “PPCD3” OR “TCF8” OR “ZFHEP” OR “ZFHX1A”) OR (“ZEB2” OR “HSPC082” OR “SIP-1” OR “SIP1” OR “SMADIP1” OR “ZFHX1B”)) AND (“cancer” OR “tumor” OR “tumour” OR “carcinoma” OR “neoplasm” OR “neoplasia” OR “adenoma” OR “sarcoma”). The reference list of each study was also manually screened in order to retrieve potentially missing studies. The literature search procedure was conducted up to September, 2016. The present study was designed, conducted and reported according to the PRISMA statement [57], as shown in Supplementary Table 1 [58].

### Study selection criteria

Two independent investigators (Wei Lu and Huihui Chen) carefully scrutinized the literatures from the initial search. Duplicated studies were first excluded, afterwards titles and abstracts were carefully skimmed, and finally full texts of potential qualified studies were reviewed. Studies were considered eligible and included if they meet the following criteria: (1) Studies were cohort studies whose patients had digestive cancers (pancreatic cancer, esophageal squamous cell carcinoma, hepatocellular carcinoma, gastric cancer, colorectal cancer, cholangiocarcinoma and hepatocellular carcinoma); (2) Expression levels of ZEB family members (ZEB1 or ZEB2) were detected in cancer tissues; (3) Studies described the association between ZEB1 or ZEB2 levels and survival outcome (overall survival or recurrence free survival or disease free survival); (4) Hazard ratio (HR) and its corresponding 95% confidence interval (CI) were available or could be calculated; (5) For studies reporting duplicated or overlapping cohorts only the most complete studies were included. Studies were excluded if they meet the following criteria: (1) Studies were not original studies such as abstracts, reviews, expert opinions, editorials or case reports; (2) Studies were based on cancer cells or animals rather than patients; (3) Patients had other types of cancer beyond digestive system; (4) Studies did not report HR and its corresponding 95% CI or they could not be calculated.

### Data extraction and quality assessment

Data extraction was conducted by two independent investigators (Wei Lu and Huihui Chen) from texts, figures and tables. The following information was extracted: first authors, year of publication, country of origin, number of patients, follow-up duration, age, specimen types, detection methods, protein/mRNA, analysis methods, endpoints, cancer types. The definitions of ZEB1 or ZEB2 high expression group were in accordance with each original study. For studies which only provided survival data in Kaplan-Meier survival curves, the software Engauge Digitizer (http://www.engauge.com/) was applied to digitize and synthesize data according to the Guyot's algorithm [59]. In this study, we combined the most fully adjusted risk estimates with their 95 % CIs. Quality assessment was performed by two investigators (Wei Lu and Huihui Chen) and consensus was reached on all items through detailed discussion. All included studies were scored according to the REMARK (reporting recommendations for tumor marker prognostic studies) guideline [60]. The scores ranged from 0 to 20 and studies with scores above 15 were considered to be of high quality.

### Data synthesis and statistical analysis

HRs and their corresponding 95% CI were extracted from each included study. Heterogeneity was determined using the chi-square test and the I^2^ test, and *p*<0.10 in combination with I^2^>50% indicated significant heterogeneity across studies. The odds ratios (ORs) and their corresponding 95% CI were also pooled to analyze the association between ZEB family expression and clinicopathological characteristics in digestive cancer patients. A fixed-effect or random-effect model was used to pool HR or OR depending on the heterogeneity analysis (if the heterogeneity was not significant, the fixed-effect model was more appropriate, otherwise the random-effect model was applied, which would provide wider 95% CI). The results were presented as forest plots. To estimate potential publication bias, Begg's funnel plots and Egger's linear regression test were performed.

Sensitivity analyses were performed to examine the impact of single study on pooled results via sequential omission of each individual study. Subgroup analyses were also conducted by cancer type, country, protein/mRNA, quality assessment score and sample size. In addition, a cumulative meta-analysis summarizing the evidence in the assessment of sample size was performed. From small to large sample size, the studies were added one at a time to pool the results sequentially. Meta-analysis was conducted using the Stata software (version 12.0; StatCorp, College Station, TX, USA). To further assess the prognostic value of ZEB1 and ZEB2 in digestive cancers, we adopted Guyot's method which derived from Kaplan-Meier survival curves a close approximation to the original individual patient data [59]. Time-to-event data from individual study were pooled to synthesize reconstructed survival curves. The log-rank test was used to compare patient survival between two groups. All the *p*-values were two-sided and *p*<0.05 was considered statistically significant unless specified.

## SUPPLEMENTARY MATERIALS TABLES





## Abbreviations

ZEB1: zinc finger E-box binding homeobox 1
ZEB2: zinc finger E-box binding homeobox 2
EMT: epithelial mesenchymal transition
OS: overall survival
DFS: disease free survival
RFS: recurrence free survival
HR: hazard ratio
OR: odds ratio
CI: confidence interval
REMARK: reporting recommendations for tumor marker prognostic studies
IHC: immunohistochemistry
qRT-PCR: quantitative real time polymerase chain reaction
