# Supplementary material for: Prognostic significance of ZEB1 and ZEB2 in digestive cancers: a cohort-based analysis and secondary analysis
Source: Oncotarget. 2017 Feb 23;8(19):31435–48. doi: 10.18632/oncotarget.15634 (PMC5458220; doi:10.18632/oncotarget.15634)
Supplement: Supplementary file 1 [file oncotarget-08-31435-s001.pdf]

Prognostic significance of ZEB1 and ZEB2 in digestive cancers: a cohort-based analysis and secondary analysis

SUPPLEMENTARY TABLE

Supplementary Table 1: PRISMA 2009 checklist

See Supplementary File 1
